# The role of cytokines in acute gastrointestinal injury: a prospective pilot study

**DOI:** 10.3389/fmed.2026.1701855

**Published:** 2026-03-04

**Authors:** Yanhua Li, Youquan Wang, Lu Ke, Xinyu Li, Lingling Bao, Feng Zhang, Hongxiang Li, Dong Zhang

**Affiliations:** 1 Department of Critical Care Medicine, The First Hospital of Jilin University, Changchun, China; 2 Department of Critical Care Medicine, Jinling Hospital, Medical School of Nanjing University, Nanjing, Jiangsu, China; 3 National Institute of Healthcare Data Science, Nanjing University, Nanjing, Jiangsu, China

**Keywords:** acute gastrointestinal injury, critical illness, cytokines, D-lactate, intensive care unit, persistent intestinal failure

## Abstract

**Objective:**

This study aimed to investigate the association between cytokines and the severity of acute gastrointestinal injury (AGI) in critically ill patients on day 3 after intensive care unit (ICU) admission.

**Methods:**

This was a single-center, prospective observational cohort study. We collected blood samples for 5 consecutive days and measured interleukin-6 (IL-6), interleukin-10 (IL-10), tumor necrosis factor-alpha (TNF-*α*), intestinal fatty acid-binding protein (I-FABP), lipopolysaccharide (LPS), and D-lactate levels in critically ill patients with AGI admitted to the ICU. The primary outcome was persistent intestinal failure, defined as AGI grades III–IV on day 3 after ICU admission. A multivariate logistic regression analysis was used to identify independent risk factors for persistent intestinal failure, and an XGBoost model was used to assess the relative importance of predictors for persistent intestinal failure. In addition, we performed a mediation analysis to evaluate the mediating role of cytokines in the association between intestinal permeability (D-lactate) and persistent intestinal failure.

**Results:**

A total of 116 patients were included in the final analysis. On day 1 after ICU admission, the levels of plasma biomarkers, such as IL-6, IL-10, TNF-*α*, I-FABP, LPS, and D-lactate, were significantly higher in the AGI III–IV group than in the AGI I–II group. These biomarkers showed a consistently decreasing trend from day 2 to day 5. The multivariate logistic regression analysis identified IL-6, IL-10, D-Lactate, and the SOFA score as independent predictors of persistent intestinal failure. Using the XGBoost algorithm, we determined the relative importance of predicting persistent intestinal failure on day 3 of ICU admission. In descending order, the key predictors were IL-6 (D1), IL-10 (D1), D-lactate (D1), the SOFA score, and sex. The mediation analysis showed that IL-6, IL-10, and TNF-*α* partially mediated the association between D-lactate levels and persistent intestinal failure. The respective proportions of their mediating effects were 73.3% for IL-6, 52.1% for IL-10, and 30.1% for TNF-*α*.

**Conclusion:**

In critically ill patients with AGI, the levels of cytokines on day 1 after ICU admission were positively associated with persistent intestinal failure during the early acute phase. Cytokines may partially mediate the relationship between intestinal injury and progression to persistent intestinal failure. Controlling the inflammatory response may represent a potential therapeutic strategy for acute gastrointestinal injury.

**Clinical trial registration:**

This study was registered with the Chinese Clinical Trial Registry on 21 February 2022 (Registration ID: ChiCTR2200056858).

## 1 Introduction

Acute gastrointestinal injury (AGI) is a prevalent complication in critically ill patients, with an incidence rate ranging from 40 to 85.5% (1–3). The severity of AGI is closely linked to the prognosis and clinical outcomes. Multiple studies have shown that higher grades of AGI are significantly associated with increased feeding intolerance and elevated mortality risk (2, 3). These observations underscore an unmet need for effective strategies to prevent or mitigate gastrointestinal dysfunction in the intensive care unit (ICU).

Available evidence suggests an association between AGI and impaired gastrointestinal barrier function (4); however, the underlying pathophysiological pathways, particularly those connecting critical illness to barrier breakdown and subsequent AGI, are not yet fully defined. It has recently been reported that intestinal immune activation and inflammatory responses might be central to the development of AGI (5, 6). Inflammatory mediators contribute to intestinal inflammation and initiate pro-inflammatory cascades, thereby exacerbating intestinal barrier dysfunction (7). In animal models, elevated levels of pro-inflammatory cytokines have been shown to adversely affect gastrointestinal function, partly by inducing apoptosis of intestinal epithelial cells; moreover, cytokine-mediated alterations in intestinal motility appear to be dose- and time-dependent (8). Some studies have identified IL-6 and IL-10 as key biomarkers of intestinal barrier dysfunction (9–12). However, the majority of available evidence is derived from mechanistic experiments or animal models. In contrast, clinical studies reporting clinically meaningful gastrointestinal outcomes are scarce and typically underpowered.

Given the close interplay between gastrointestinal dysfunction, gut barrier disruption, and systemic inflammation in critical illness, early identification of patients at risk of developing persistent gastrointestinal dysfunction is clinically relevant, as it may guide monitoring intensity and supportive interventions, including nutritional strategies. The AGI grading system (13) is currently the most widely accepted tool for evaluating gastrointestinal dysfunction severity in critically ill patients. Nevertheless, it remains unclear whether elevated cytokine levels are independently associated with subsequent persistent gastrointestinal failure and whether cytokines mediate the relationship between increased intestinal mucosal permeability and progression to persistent intestinal dysfunction. Addressing these gaps could improve risk stratification and inform potential therapeutic targets in AGI.

## 2 Methods

### 2.1 Study design

This was a single-center, prospective observational study. This study was conducted at the Department of Critical Care Medicine, First Hospital of Jilin University, Changchun, Jilin, China, from 18 September 2022 to 18 September 2023. This study aimed to monitor the plasma levels of key biomarkers, such as IL-6, IL-10, TNF-*α*, I-FABP, D-lactate, and LPS, during the acute phase (within the first 5 days after ICU admission) in critically ill patients with AGI. The primary clinical outcome was persistent intestinal failure, defined as AGI grades III–IV on ICU day 3. We described the temporal dynamics of these biomarkers and evaluated their association with AGI severity. Additionally, we conducted a comprehensive analysis of the risk factors associated with persistent intestinal failure and quantified the relative importance of each factor. Finally, a mediation analysis was performed to clarify the role of cytokines between intestinal permeability (D-lactate) and persistent intestinal failure.

This study was approved by the Ethics Committee of the First Hospital of Jilin University (Approval Number: AF-IRB-032-06) and adhered to the ethical guidelines outlined in the World Medical Association’s Declaration of Helsinki. The study was registered in the WHO-accredited Chinese Clinical Trial Registry on 21 February 2022 (Registration ID: ChiCTR2200056858).

### 2.2 Patient selection and grouping

Patients were enrolled according to the following inclusion criteria: age ≥18 years and a diagnosis of AGI based on the definition provided by the European Society of Intensive Care Medicine (ESICM) (13), with specific grading criteria provided in Supplementary Table S1. In addition, patients or their authorized representatives were asked to provide informed consent for participation. The exclusion criteria comprised pre-existing Crohn’s disease or ulcerative colitis, short bowel syndrome, pregnancy or lactation, an anticipated hospital stay of less than 5 days, and refusal to participate in the study.

In accordance with our prior investigations (14) and the latest Chinese guidelines (15), AGI severity was stratified into two categories: grades I–II, indicating intestinal dysfunction, and grades III–IV, indicating intestinal failure.

### 2.3 Data collection and clinical evaluation

For each enrolled patient, 2 mL of blood was collected daily from the day of enrollment through day 5 after ICU admission. Blood samples were immediately centrifuged, and the resulting plasma was stored at −80 °C. Plasma analyses were performed within 2 weeks using enzyme-linked immunosorbent assay (ELISA) kits (R&D Systems, Minneapolis, United States). Human specimens were obtained from the Department of Biobank, Division of Clinical Research, First Hospital of Jilin University, Changchun, Jilin, China.

Based on our previous study (4), the reference plasma levels of I-FABP and LPS (pg/mL) and D-lactate (μmol/L) in healthy individuals were 31.32 (24.54–34.87), 2.65 (1.17–3.45), and 8.21 (3.23–10.37), respectively (median [IQR]). Furthermore, we measured the plasma levels of IL-6, IL-10, and TNF-*α* in 50 healthy individuals recruited from a health checkup center. The corresponding reference values were 2.64 (1.23–4.01), 5.68 (3.23–7.04), and 6.45 (2.36–9.34) pg./mL, respectively [median (IQR); Supplementary Table S2]. The healthy reference samples were processed and assayed using procedures comparable to those used for patient samples in the same laboratory in the present study.

The following clinical data were collected: demographic characteristics, including age, sex, primary diagnosis, AGI grade, intra-abdominal pressure (IAP), the Acute Physiology and Chronic Health Evaluation (APACHE) II score, and the Sequential Organ Failure Assessment (SOFA) score; information on whether mechanical ventilation and continuous renal replacement therapy (CRRT) were performed; and clinical outcomes, including 7-day mortality, 28-day mortality, and length of hospital stay.

### 2.4 Sample size

This exploratory pilot study was not designed for formal hypothesis testing; therefore, no power-based sample size calculation was performed. Instead, for the multivariable logistic regression analysis, the target sample size was based on the anticipated number of outcome events relative to model complexity, expressed as events per variable (EPV) (16). With four prespecified candidate predictors, we targeted 8–10 EPV (approximately 32–40 outcome events) to support exploratory multivariable analyses and preliminary effect size estimation. Based on the findings of our team’s systematic review and meta-analysis (2), the AGI grade III–IV event rate was approximately 35%. Therefore, a total sample size of 91–114 patients was expected to yield approximately 32–40 events.

### 2.5 Statistical analysis

The distribution of continuous variables was assessed using the Kolmogorov–Smirnov test. Quantitative variables with a normal distribution are reported as mean ± standard deviation (SD) and were compared using Student’s t-test. For day-specific between-group comparisons across ICU days 1–5 involving six biomarkers (30 comparisons in total), *p*-values were adjusted for multiple testing using the Benjamini–Hochberg false discovery rate (BH-FDR) procedure. Both unadjusted p-values and BH-FDR-adjusted P–values were reported. In contrast, non-normally distributed quantitative variables are expressed as median and interquartile range and analyzed using the Mann–Whitney U test. Categorical variables are presented as absolute numbers and percentages and were compared using the χ^2^ test or Fisher’s exact test, as appropriate.

To identify independent risk factors for persistent intestinal failure, a multivariate logistic regression analysis was performed using a backward stepwise approach. Variables with a significance level of *p* < 0.1 in univariate analyses were included in the model. Collinearity among covariates was evaluated using Spearman’s correlation and the Belsley collinearity test. Receiver operating characteristic (ROC) curves and the area under the ROC curve (AUC) with 95% confidence intervals (CI) were used to evaluate the discriminative ability of individual predictors measured on ICU day 1 for persistent intestinal failure. For each predictor, the optimal cutoff value was determined by maximizing the Youden index (J = sensitivity + specificity − 1). In the combined multivariable model, predicted probabilities were used to construct ROC curves and estimate AUC. Furthermore, the XGBoost algorithm was used to quantify the relative importance of each risk factor in persistent intestinal failure.

We performed regression-based mediation analyses to assess whether inflammatory cytokines measured on ICU day 1 mediate the association between impaired intestinal permeability and subsequent persistent intestinal failure. Plasma D-lactate on ICU day 1 was defined as the exposure (X), cytokines on ICU day 1 (IL-6, TNF-*α*, and IL-10) were defined as candidate mediators (M), and persistent intestinal failure—defined as AGI grades III–IV on ICU day 3—was defined as the outcome (Y). Guided by a prespecified causal framework (directed acyclic graph (DAG)), we decomposed the total effect of D-lactate on AGI grades III–IV (c) into the direct effect (c′) and the indirect effect through each cytokine (a × b), with the proportion mediated calculated as (a × b)/c. Uncertainty was quantified using a bias-corrected and accelerated (BCa) bootstrap with 10,000 resamples to derive 95% confidence intervals. Age and SOFA score were included as core baseline severity covariates; residual confounding may remain. Each cytokine was evaluated in a separate single-mediator model unless otherwise specified.

All statistical analyses were performed using SPSS for Mac version 26 (SPSS Inc., Chicago, IL, United States) and R v4.3.1 (R Foundation for Statistical Computing, Vienna, Austria) with RStudio v1.0.136 (RStudio Inc., Boston, MA, USA). A two-tailed *p*-value of < 0.05 was considered statistically significant.

## 3 Results

### 3.1 Participant selection and study flow

**Figure 1:**
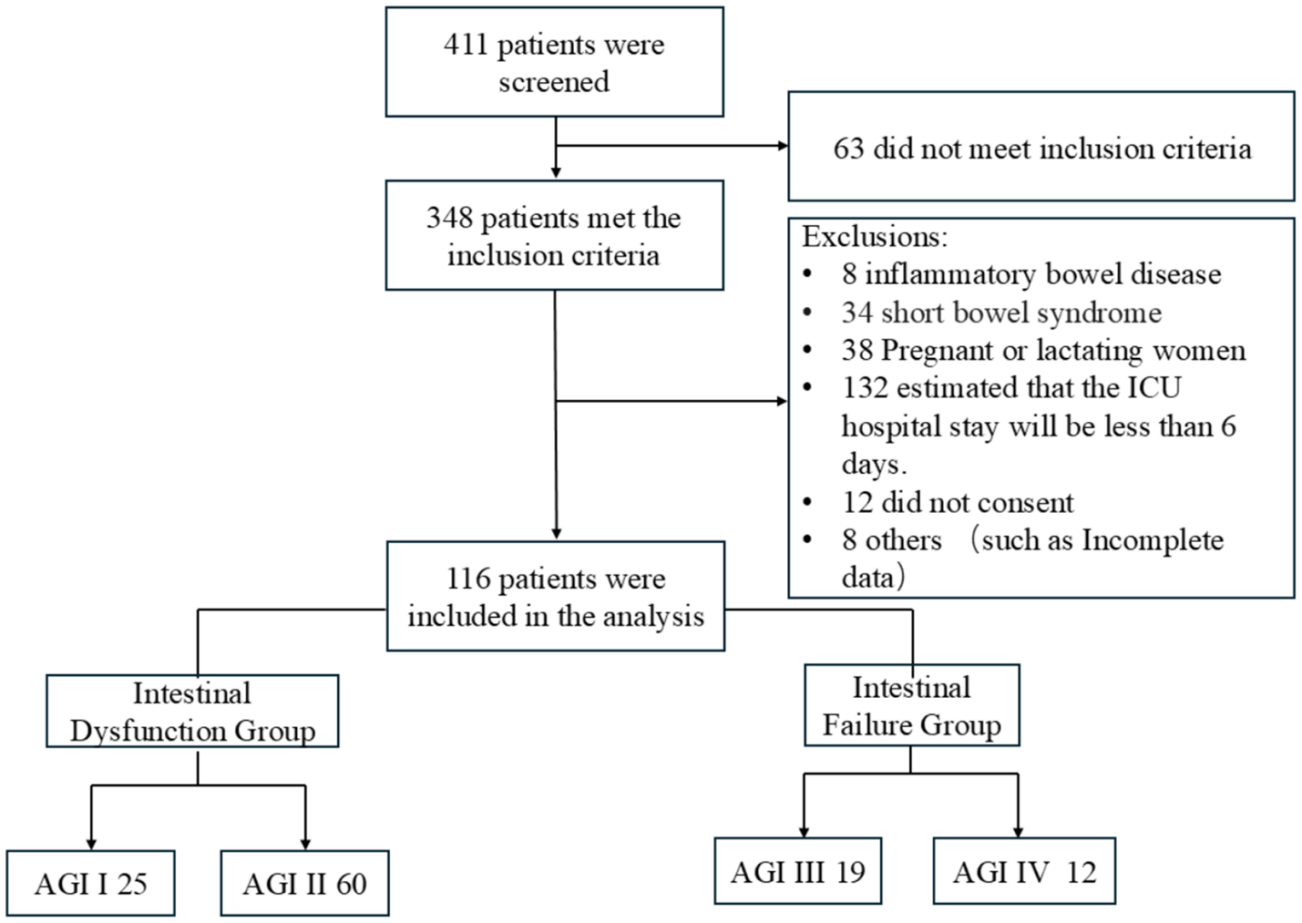
Study flow diagram of patient screening, eligibility assessment, and inclusion. AGI, acute gastrointestinal injury; ICU, intensive care unit.

A total of 411 patients were initially screened (Figure 1). Of these, 63 were excluded because they did not meet the inclusion criteria, and 232 were excluded due to exclusion criteria or other reasons. Consequently, 116 patients were eligible and included in the final analysis. Patients were classified into two primary groups according to the grading criteria. The intestinal dysfunction group (AGI grades I–II) comprised patients with AGI grade I (*n* = 25) and grade II (*n* = 60), whereas the intestinal failure group (AGI grades III–IV) included those with AGI grade III (*n* = 19) and grade IV (*n* = 12).

### 3.2 Baseline characteristics of the study population

A total of 116 patients were enrolled and classified into two distinct groups based on AGI severity: the AGI I–II group (intestinal dysfunction), comprising 85 patients, and the AGI III–IV group (intestinal failure), comprising 31 patients. The baseline characteristics of these groups are summarized in Table 1.

**Table 1:** Baseline characteristics of patients with AGI I–II and AGI III–IV groups.

Baseline characteristics	AGI I–II group (*n* = 85)	AGI III–IV group (*n* = 31)	*p*
Age (median, IQR)	66 (45,81)	74 (51.5,81)	0.32
Male *n* (%)	62 (72.9%)	28 (90.3%)	0.05
APACHE II (median, IQR)	19 (12,22)	22 (18,29)	<0.001*
SOFA (median, IQR)	5 (4,8)	6 (4.5,11.5)	0.05
Primary AGI *n* (%)	28 (32.9%)	13 (41.9%)	0.37
Diagnose *n* (%)			
Shock	2 (2.4%)	3 (9.7%)	
Sepsis	11 (12.9%)	4 (12.9%)	
Respiratory failure	29 (34.1%)	13 (41.9%)	
Trauma	10 (11.8%)	0(0%)	
Postoperation	7 (8.2%)	2 (6.5%)	
Cardiac arrest	3 (3.5%)	2 (6.5%)	
SAP	18 (21.2%)	7 (22.6%)	
Renal failure	5 (5.9%)	0 (0%)	
MV *n* (%)	67 (78.8%)	26 (83.9%)	0.55
CRRT *n* (%)	14 (16.5%)	3 (9.7%)	0.55
7-day mortality *n* (%)	2 (2.4%)	3 (9.7%)	0.12
28-day mortality *n* (%)	9 (10.7%)	9 (29.0%)	0.02*
LOS (median, IQR)	17 (9,24)	18 (8,28)	0.96

AGI, acute gastrointestinal injury; IQR, interquartile range; APACHE II, Acute Physiology and Chronic Health Evaluation II; SOFA, Sequential Organ Failure Assessment; SAP, severe acute pancreatitis; MV, mechanical ventilation; CRRT, continuous renal replacement therapy; LOS, length of stay;*, *p* < 0.05.

### 3.3 Comparison of cytokine levels between the AGI groups

**Figure 2:**
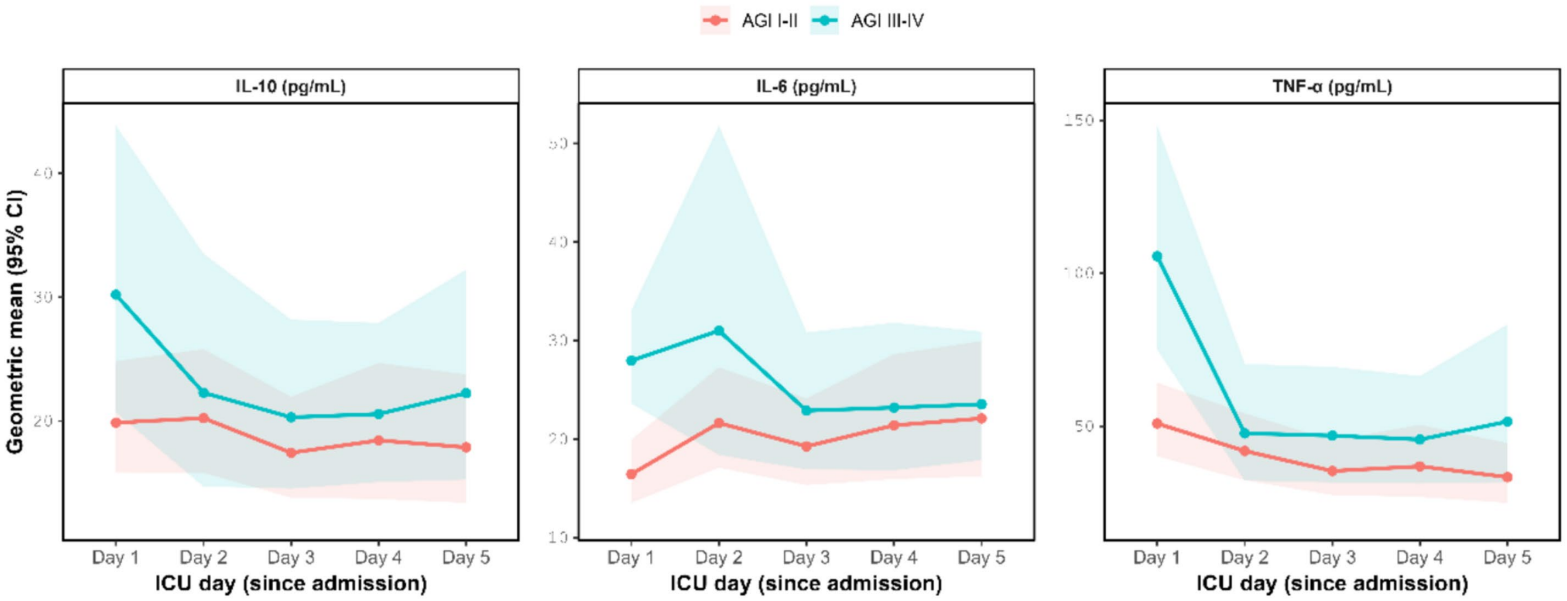
Cytokine trajectories by the AGI group during the first 5 ICU days. Points and shaded areas represent geometric means and 95% CIs estimated from linear mixed models on the log(value+1) scale and back-transformed. AGI, acute gastrointestinal injury; IL, interleukin; TNF, tumor necrosis factor.

On day 1, cytokine concentrations were higher in the AGI III–IV group than in the AGI I–II group, with between-group differences reaching statistical significance. In contrast, no statistically significant between-group differences were observed from day 2 to day 5. These conclusions remained unchanged after adjusting for multiple comparisons across days 1–5 using the Benjamini–Hochberg false discovery rate procedure, and significance was interpreted primarily based on the adjusted *p*-values (Table 2), and the temporal trends in cytokine levels over the study period are illustrated in Figure 2.

**Table 2:** Comparison of cytokine levels between AGI I–II and AGI III–IV groups.

Study day (D1–D5)	AGI I–II group (*n* = 85)	AGI III–IV group (*n* = 31)	*p*	Adjusted P (BH-FDR)
IL-6 (pg/ml)				
D1	20.43 (4.19, 27.91)	31.76 (29.35, 33.80)	0.001	0.008*
D2	17.17 (14.36, 24.47)	19.22 (16.10, 21.07)	0.141	0.285
D3	17.79 (13.67, 20.18)	18.35 (16.46, 20.75)	0.268	0.371
D4	16.95 (13.54, 20.31)	19.00 (15.61, 20.09)	0.199	0.358
D5	16.41 (13.72, 21.05)	19.33 (18.81, 19.98)	0.107	0.236
IL-10 (pg/ml)				
D1	18.26 (14.13, 21.38)	21.23 (19.30, 23.62)	0.004	0.022*
D2	13.98 (12.13, 16.78)	16.28 (13.42, 18.40)	0.358	0.432
D3	14.15 (11.40, 16.18)	15.46 (13.82, 17.08)	0.419	0.489
D4	14.34 (11.72, 16.75)	15.77 (13.12, 17.90)	0.290	0.388
D5	14.31 (11.56, 18.93)	16.38 (15.36, 17.08)	0.067	0.226
TNF-α (pg/ml)				
D1	68.57 (31.55, 87.79)	92.69 (82.69, 111.36)	<0.001	0.008*
D2	28.10 (23.42, 32.73)	32.31 (26.47, 34.93)	0.312	0.392
D3	28.78 (22.93, 33.83)	31.68 (27.86, 34.44)	0.261	0.371
D4	27.92 (22.80, 36.18)	31.50 (25.97, 33.290)	0.264	0.371
D5	27.83 (22.50, 39.73)	32.63 (30.91, 33.13)	0.051	0.192

AGI, acute gastrointestinal injury; IQR, interquartile range; IL, interleukin; TNF, tumor necrosis factor; *p<0.05.

### 3.4 Comparison of biomarkers of intestinal injury between the AGI groups

**Figure 3:**
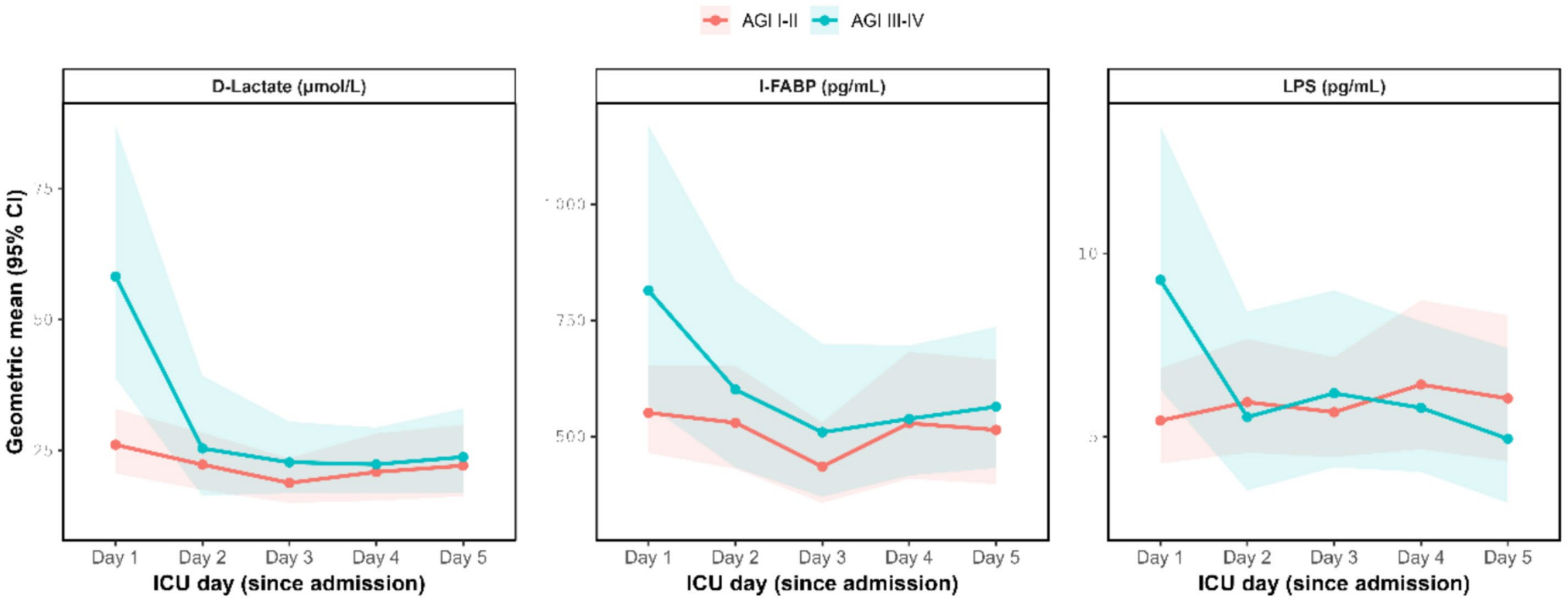
Intestinal injury biomarker trajectories by the AGI group during the first 5 ICU days. Points and shaded areas represent geometric means and 95% CIs estimated from linear mixed models on the log(value+1) scale and back-transformed. AGI, acute gastrointestinal injury; I-FABP, intestinal fatty acid-binding protein; LPS, lipopolysaccharide; ICU, intensive care unit.

On day 1, biomarkers of intestinal injury, including I-FABP, D-lactate, and LPS, were higher in the AGI III–IV group than in the AGI I–II group, with between-group differences reaching statistical significance. In contrast, no significant differences were observed from day 2 to day 5. These conclusions remained unchanged after adjusting for multiple comparisons across days 1–5 using the Benjamini–Hochberg false discovery rate procedure, with significance interpreted primarily based on the adjusted *p*-values (Table 3). The trends in biomarker changes over time are illustrated in Figure 3.

**Table 3:** Comparison of intestinal injury biomarker levels between AGI I–II and AGI III–IV groups.

Study day (D1–D5)	AGI I–II group (*n* = 85)	AGI III–IV group (*n* = 31)	*p*	Adjusted P (BH-FDR)
I-FABP (pg/mL)				
D1	553.90 (447.63, 649.42)	653.03 (564.19, 771.28)	0.002	0.013*
D2	452.38 (347.07, 513.67)	488.43 (395.12, 527.28)	0.089	0.236
D3	422.88 (347.08, 485.36)	472.59 (421.10, 518.94)	0.092	0.236
D4	433.31 (321.21, 562.03)	479.89 (393.66, 531.18)	0.202	0.359
D5	414.03 (337.37, 545.34)	494.51 (431.34, 538.53)	0.103	0.123
D-lactate (μmol/L)				
D1	40.56 (15.96, 60.10)	57.71 (49.69, 61.90)	0.001	0.008*
D2	16.92 (13.87, 20.48)	19.65 (15.97, 22.29)	0.272	0.371
D3	16.76 (13.40, 19.42)	19.08 (16.86, 21.04)	0.111	0.236
D4	17.53 (13.63, 21.12)	19.23 (15.86, 21.38)	0.256	0.371
D5	16.72 (14.24, 21.98)	19.65 (18.64, 20.93)	0.089	0.236
LPS (pg/mL)				
D1	5.26 (3.66, 7.44)	7.17 (6.78, 7.44)	0.002	0.013*
D2	4.67 (2.76, 6.95)	3.35 (3.16, 3.75)	0.528	0.597
D3	4.46 (2.61, 7.67)	3.33 (3.04, 4.24)	0.879	0.916
D4	4.23 (2.68, 6.95)	3.38 (3.16, 4.00)	0.958	0.961
D5	4.70 (2.66, 7.11)	3.28 (3.14, 4.77)	0.795	0.857

AGI, Acute gastrointestinal injury; IQR, Interquartile range; I-FABP, Intestinal fatty acid-binding protein; LPS, Lipopolysaccharide; *p < 0.05.

### 3.5 Logistic regression analysis of risk factors for persistent intestinal failure

**Figure 4:**
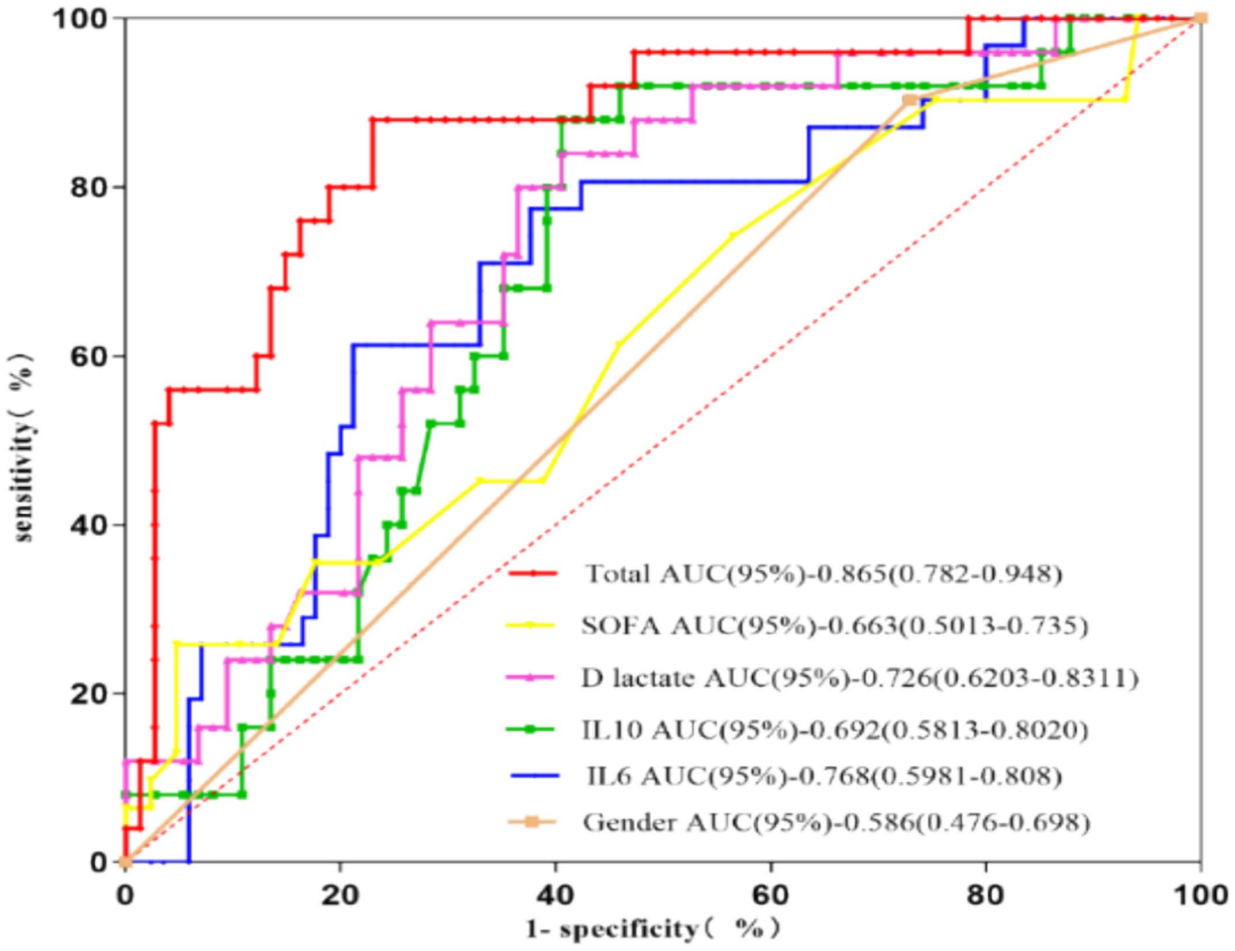
ROC curve and AUC for predicting the occurrence of persistent intestinal failure. SOFA, Sequential Organ Failure Assessment; IL, interleukin; AUC, area under the curve.

Variables with a *p*-value of < 0.10 in univariate analyses were entered into multivariable logistic regression analyses. After collinearity assessment (Supplementary Figure S2), sex, the SOFA score, IL-6 (D1), IL-10 (D1), and D-lactate (D1) were included in the final model. Higher SOFA score, IL-6 (D1), IL-10 (D1), and D-lactate (D1) were independently associated with persistent intestinal failure (Table 4). ROC analysis showed an AUC of 0.865 (95%CI: 0.782–0.948) for predicting persistent intestinal failure (Figure 4). The optimal cutoff value was determined by maximizing the Youden index, and the corresponding sensitivity, specificity, and Youden’s J are reported in Table 4.

**Table 4:** Multivariable logistic regression and ROC analyses of candidate predictors for persistent intestinal failure.

Predictors	OR (95%CI)	*p*	AUC (95%CI)	Optimal cutoff	Sens	Spec	Youden’s J
Sex	2.94 (0.57–15.30)	0.199	0.59 (0.48–0.70)	1.5	0.90	0.39	0.30
SOFA	1.15 (1.01–1.32)	0.038	0.66 (0.50–0.74)	12.5	0.30	0.95	0.21
IL-6 (D1)	1.14 (1.05–1.23)	0.001	0.77 (0.60–0.81)	20.38 pg./mL	0.81	0.73	0.54
IL-10 (D1)	1.04 (1.02–1.07)	0.001	0.69 (0.58–0.80)	17.15 pg./mL	0.92	0.73	0.65
D-lactate (D1)	1.01 (1.00–1.02)	0.021	0.73 (0.62–0.83)	19.18 μmol/L	0.92	0.69	0.61

OR, Odds ratio; CI, Confidence interval; SOFA, Sequential Organ Failure Assessment; IL, Interleukin; AGI, Acute gastrointestinal injury. Sens, Sensitivity; Spec, Specificity.

**Figure 5:**
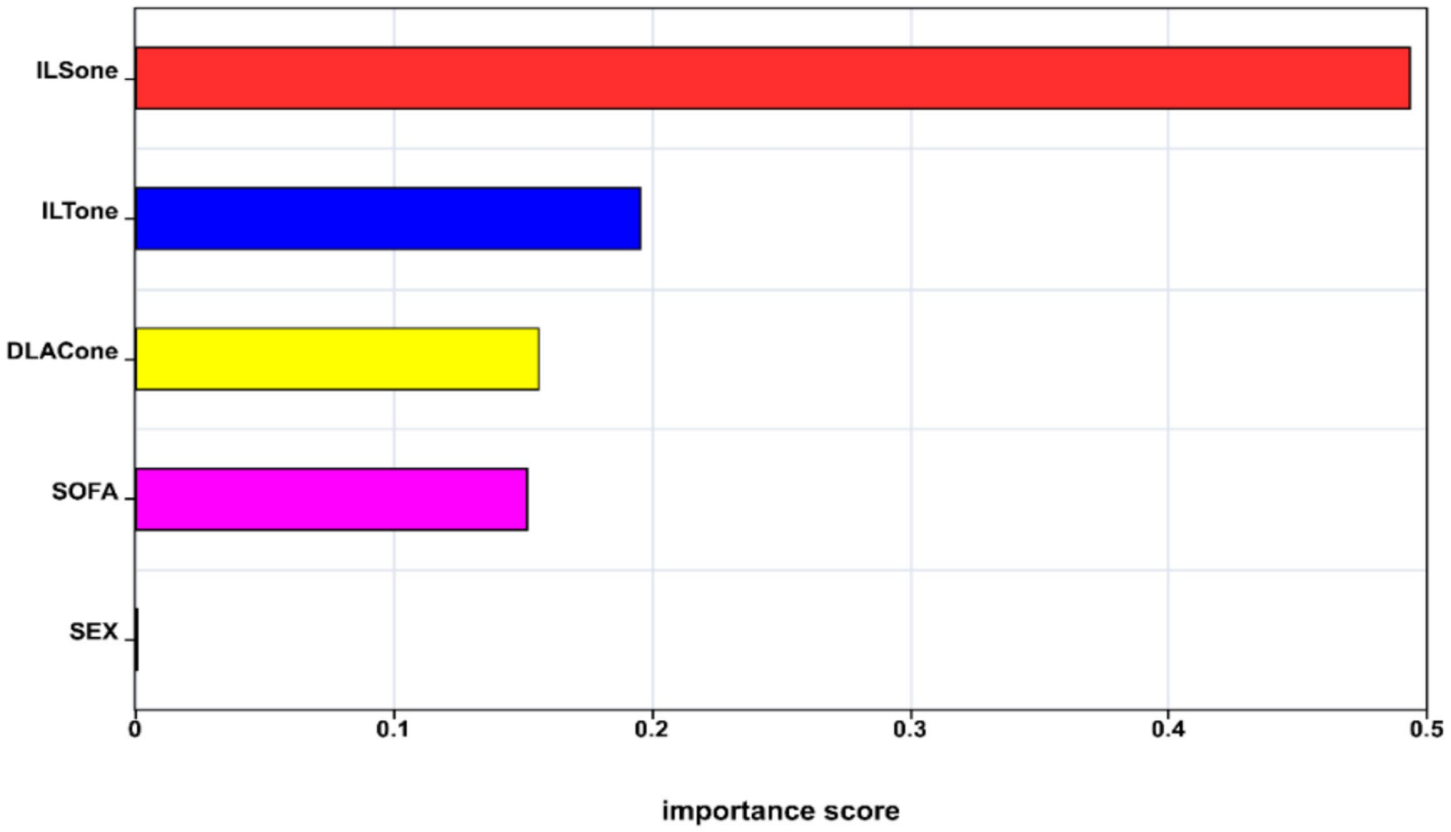
Importance scores of variables in the logistic regression analysis. ILSONE, interleukin-6 on ICU day 1; ILTONE, interleukin-10 on ICU day 1; DLACONE, D-lactate on ICU day 1; SOFA, Sequential Organ Failure Assessment.

XGBoost feature importance ranked IL-6 (D1), IL-10 (D1), D-lactate (D1), the SOFA score, and sex as the leading predictors (Figure 5).

### 3.6 Mediation analysis of the association between intestinal permeability and persistent intestinal failure

**Figure 6:**
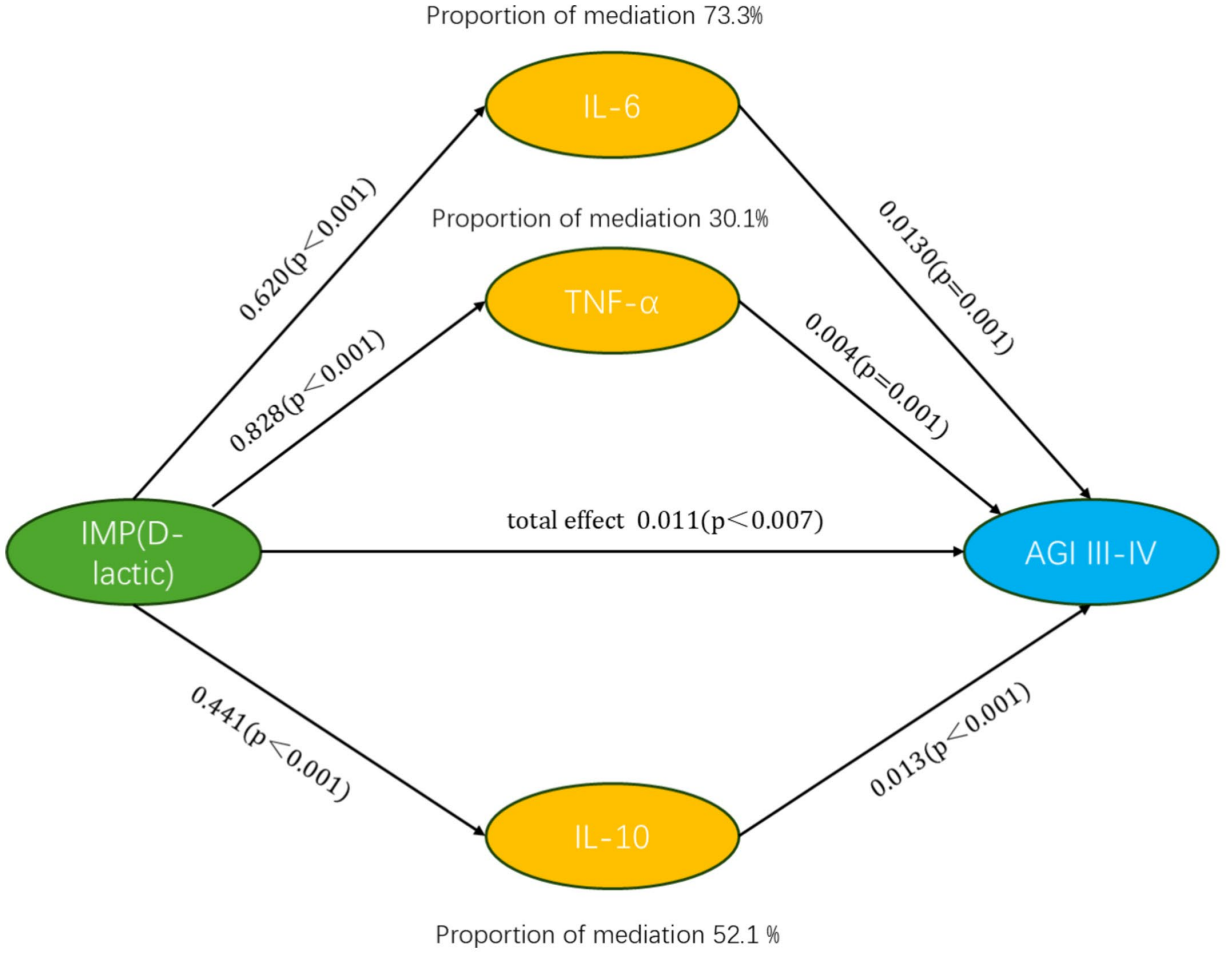
Mediation pathway with estimated effects Path coefficients *β* and *p*-values are shown for the associations between D-lactate (X), IL-6, IL-10, and TNF-*α* (M), and persistent intestinal failure (AGI grades III–IV on ICU day 3) (Y). The total effect and the proportion mediated are reported based on bootstrap 95% CIs. Each cytokine was tested in a separate single-mediator model. IMP, intestinal mucosal permeability; AGI, acute gastrointestinal injury; IL, interleukin; TNF, tumor necrosis factor.

The results indicated that cytokines IL-6, IL-10, and TNF-*α* exhibited significant mediating effects on the relationship between intestinal mucosal permeability (D-lactate) and persistent intestinal failure (AGI grades III–IV on ICU day 3), accounting for 73.3, 52.1, and 30.1% of the total effect, respectively. Detailed results are shown in Figure 6 and Supplementary Table S3.

## 4 Discussion

In this study, several key findings emerged. First, compared with the AGI I–II group, patients who developed persistent intestinal failure had significantly higher levels of IL-6, IL-10, and TNF-*α* on ICU day 1, along with elevated intestinal injury-related biomarkers (I-FABP, LPS, and D-lactate), indicating that an early inflammatory surge and impaired gut barrier function are closely associated with progression to severe gastrointestinal dysfunction. Second, the multivariable logistic regression analysis identified IL-6, IL-10, the SOFA score, and D-lactate on ICU day 1 as independent factors associated with persistent intestinal failure, highlighting the potential value of inflammatory burden and gut barrier markers for early risk stratification. Third, mediation analyses further suggested that cytokines partially mediated the association between D-lactate (a marker consistent with intestinal permeability) and persistent intestinal failure, providing supportive evidence for an inflammation-related pathway linking gut barrier dysfunction to sustained severe AGI.

Based on the conclusions of our previous study (14), classifying AGI into two categories—gastrointestinal dysfunction (AGI I–II group) and gastrointestinal failure (AGI III–IV group)—has been shown to be more effective in predicting prognosis than the traditional four-grade AGI classification system. This classification approach aligns with the latest guidelines in China (15). Therefore, in the present study, we adopted this two-tiered classification, categorizing patients into the AGI I–II group and the AGI III–IV group.

In our cohort, IL-6, IL-10, and TNF-*α* levels on ICU day 1 were significantly higher in patients with AGI grades III–IV than in those with grades I–II, suggesting an association between an amplified cytokine response and more severe gastrointestinal dysfunction. This finding is consistent with the observations of Wand et al. (17). Mechanistically, inflammatory responses can increase vascular permeability, leading to fluid extravasation, intestinal interstitial edema, and local hypoperfusion, thereby aggravating gastrointestinal dysfunction and potentially contributing to its persistence (18). The underlying mechanisms for this association may involve several pathways (12, 19, 20): (1) elevated cytokine levels can modulate intestinal permeability by altering the expression of tight junction proteins, including claudin-2, claudin-5, junctional adhesion molecule A (JAM-A), occludin, and zonula occludens-1 (ZO-1); (2) pro-inflammatory cytokines can suppress intestinal epithelial cell regeneration while promoting apoptosis via a toll-like receptor 4 (TLR4)-dependent mechanism; (3) cytokine-induced alterations in the mucus layer, including reduced thickness, diminished luminal coverage, and impaired adherence, further compromise gut barrier integrity; and (4) myosin light-chain kinase (MLCK) transcription is upregulated via lymphotoxin-like inducible protein (LIGHT). Increased MLCK activation is associated with compromised intestinal barrier function and increased permeability. A transient rise in cytokines suggests an early inflammatory peak in AGI. We focused on cytokines measured on ICU day 1 because they represent the earliest systemic inflammatory response after ICU admission and are most relevant for early risk stratification. Importantly, measurements taken on day 1 precede substantial confounding by subsequent ICU interventions and evolving complications, making them more suitable as early predictors rather than as late correlates. Intestinal injury remains difficult to treat due to persistent inflammation and limited regenerative capacity (21). Early identification and targeted modulation of inflammation might help attenuate subsequent worsening of gastrointestinal function. Notably, previous mouse studies (12) have shown that anti-IL-6 therapy can attenuate intestinal permeability by downregulating claudin-2 expression. In line with this finding, recent preclinical evidence (22) also suggests that suppressing NF-κB signaling, a central pro-inflammatory transcriptional pathway, can alleviate intestinal injury and preserve barrier function.

In our study, AGI was assessed on ICU day 3 to distinguish sustained intestinal failure from transient early dysfunction. During the first 48–72 h, AGI grading may fluctuate with resuscitation, hemodynamic stabilization, sedation/analgesia, and other supportive interventions. By day 3, supportive care has generally stabilized, and AGI assessment is more reflective of persistent dysfunction. Our results showed that early elevation of cytokines on ICU day 1 was associated with persistent intestinal failure, suggesting a potentially modifiable early window for prevention. We report Youden-derived cutoff values as exploratory reference points; however, these thresholds were derived from a single-pilot cohort and have not been externally validated; therefore, they should not be used as definitive bedside decision rules. In practice, a high-risk biomarker profile (elevated cytokines together with D-lactate and the SOFA score) may be used for risk stratification, prompting closer gastrointestinal monitoring and earlier optimization of supportive care. Prospective studies are needed to validate these cutoffs and to determine whether biomarker-guided interventions can improve outcomes.

Interestingly, despite its widely recognized anti-inflammatory properties, IL-10 was also significantly elevated in the AGI III–IV group compared to the AGI I–II group. This suggests a compensatory feedback mechanism aimed at mitigating tissue damage caused by excessive pro-inflammatory responses. However, while IL-10 may exert short-term protective effects, its prolonged elevation is often associated with immunosuppression. Increased IL-10 levels may not only reflect a negative feedback response to excessive inflammation but may also contribute to immune dysfunction, potentially exacerbating disease progression in critically ill patients.

In the present study, cytokine levels decreased significantly from ICU day 2 to day 5, with no significant differences observed between the AGI I–II and AGI III–IV groups. This may indicate that the initial inflammatory surge attenuates within the first few days, potentially due to clinical interventions that stabilize systemic inflammation. Alternatively, intrinsic regulatory mechanisms, including homeostatic feedback via IL-10 and other anti-inflammatory pathways, may contribute to the normalization of cytokine levels across different AGI severities. The lack of significant cytokine differences from ICU day 2 to day 5 may imply that, after the early phase, management should prioritize maintaining a balanced immune response rather than complete suppression, as overly aggressive anti-inflammatory strategies could increase the risk of complications such as nosocomial infections. Bamias et al. (23) have previously proposed that cytokines serve as central mediators of immune responses at the intestinal mucosa, with the cytokine network determining whether temporary insults result in rapid mucosal healing or trigger a deleterious pro-inflammatory cascade leading to tissue damage. The temporal dynamics observed in our study reinforce the critical role of timing in the management of AGI.

Inflammation is believed to be one of the key mechanisms underlying intestinal barrier dysfunction (24). To further assess intestinal barrier function, we measured three biomarkers daily (4, 5, 15, 25)—LPS, D-lactate, and I-FABP—over the first 5 days after ICU admission. On ICU day 1, the levels of these biomarkers were significantly higher in the AGI III–IV group than in the AGI I–II group, which is consistent with previous studies (4, 5, 26). However, these between-group differences were no longer significant from ICU day 2 onward, suggesting that early intestinal injury and permeability impairment are closely associated with AGI severity and may attenuate during the early ICU course, potentially influenced by clinical management. Taken together, these findings highlight the importance of early detection and individualized management of inflammation and intestinal injury in patients with AGI to improve clinical outcomes.

AGI grade is associated with poor prognosis (2, 27, 28). Consequently, early detection of intestinal failure and prompt intervention are critical for improving patient prognosis. To evaluate the impact of various factors on persistent intestinal failure, we performed a multivariable logistic regression analysis. The results showed that IL-6 (D1), IL-10 (D1), D-lactate (D1), and the SOFA score were independent predictors of persistent intestinal failure. Furthermore, the ROC curve analysis demonstrated that our model had robust sensitivity and specificity for predicting persistent intestinal failure, with an AUC (95% CI) of 0.865 (0.782–0.948). Notably, D-lactate rather than I-FABP was retained in the multivariable logistic regression model. This is consistent with findings from Reintam Blaser et al. (29). D-lactate has been proposed as a marker of intestinal mucosal injury and has been reported to correlate with intestinal permeability (14, 25–27). Increased permeability may facilitate the translocation of luminal microbial products, thereby amplifying systemic inflammation (5). In turn, barrier failure-associated local and systemic inflammatory/immune activation can further exacerbate mucosal injury and delay epithelial restitution, establishing a self-perpetuating feed-forward loop that may contribute to persistent intestinal failure (30). This framework also provides a biological rationale for the prominent predictive value of early cytokine responses in our models.

In addition, feature importance from the XGBoost model highlighted IL-6 (D1), IL-10 (D1), D-lactate (D1), the SOFA score, and sex as the most influential contributors to predicting persistent intestinal failure, with cytokines demonstrating greater relative importance than D-lactate. Consistently, our mediation analysis indicated that early cytokine responses partially accounted for the association between D-lactate and persistent intestinal failure. Together, these findings support a potential inflammatory pathway linking increased intestinal permeability to persistent intestinal failure, and they motivate future studies evaluating anti-inflammatory or immunomodulatory interventions.

Several strengths of our study merit highlighting. First, few previous studies have examined the relationship between early ICU inflammatory markers and subsequent persistent intestinal failure; our findings help address this gap and provide preliminary evidence relevant to the clinical management of AGI. Second, a key strength of our investigation was the serial, concurrent measurement of cytokines and intestinal injury markers during the first 5 days of ICU admission. This design enabled us to delineate the temporal dynamics of cytokine levels and intestinal injury markers during the acute phase—a facet that has been seldom explored in previous studies. Third, our results support the potential use of biomarkers such as IL-6, IL-10, and D-lactate, together with the SOFA score, to aid risk stratification and more individualized management of AGI severity. In addition, we explored the potential mediating role of cytokines in the association between intestinal barrier injury (proxied by D-lactate) and persistent intestinal failure, providing further mechanistic support for an inflammation-related pathway. In conclusion, our study not only underscores the importance of the gut in critical illness and organ dysfunction but also supports a shift toward pathophysiology-guided intestinal-protection strategies, thereby potentially improving outcomes in critically ill patients.

Several limitations of this study warrant consideration. First, we used the AGI scoring system recommended by the 2012 ESICM Working Group. This scoring system is complex and partly subjective (29). It is not based on a single objective symptom but includes a subjective assessment of the general development of a patient’s condition. Moreover, gastrointestinal function assessments may be influenced by dysfunction in other organs. Second, the relatively small sample size—attributable to the requirement for daily measurements of multiple biomarkers during the first 5 days of ICU admission—may limit the generalizability of our findings. Third, our investigation focused solely on three inflammatory markers; however, additional biomarkers such as IL-1β, interferon-*γ* (IFN-γ), and HMGB1 might also play critical roles in the AGI pathophysiology. Future studies should include a broader panel of biomarkers to better characterize the underlying inflammatory dynamics. Finally, the observed temporal convergence of cytokine levels raises concerns regarding the long-term implications of the initial inflammatory differences. It remains to be determined whether the early inflammatory surge in patients with AGI grades III–IV will ultimately lead to poorer long-term outcomes, a hypothesis that merits further investigation through extended follow-up studies.

## 5 Conclusion

In critically ill patients with AGI, higher cytokine levels on ICU day 1 were associated with the development of persistent intestinal failure during the early acute phase. Cytokines partially mediated the relationship between intestinal permeability and persistent intestinal failure. Together, these findings support an inflammation-related pathway linking intestinal barrier dysfunction to severe AGI and highlight inflammatory responses as potential targets for future mechanistic and interventional studies.

## Data Availability

The original contributions presented in the study are included in the article/Supplementary material, further inquiries can be directed to the corresponding authors.

## References

[1] Bachmann KF Jenkins B Asrani V Bear DE Bolondi G Boraso S . Core outcome set of daily monitoring of gastrointestinal function in adult critically ill patients: a modified Delphi consensus process (Cosmogi). Crit Care. (2024) 28:420. doi: 10.1186/s13054-024-05192-8, 39695807 PMC11654350

[2] Zhang D Li Y Ding L Fu Y Dong X Li H. Prevalence and outcome of acute gastrointestinal injury in critically ill patients: a systematic review and meta-analysis. Medicine (Baltimore). (2018) 97:e12970. doi: 10.1097/md.0000000000012970, 30412121 PMC6221717

[3] Wang Y Li Y Zhang Y Wang H Li Y Zhang L . Development and validation of a nomogram for predicting 28-day mortality in critically ill patients with acute gastrointestinal injury: prospective observational study. Front Nutr. (2024) 11:1469870. doi: 10.3389/fnut.2024.1469870, 39449820 PMC11499162

[4] Li H Chen Y Huo F Wang Y Zhang D. Association between acute gastrointestinal injury and biomarkers of intestinal barrier function in critically ill patients. BMC Gastroenterol. (2017) 17:45. doi: 10.1186/s12876-017-0603-z, 28356059 PMC5372314

[5] Habes QLM Van Ede L Gerretsen J Kox M Pickkers P. Norepinephrine contributes to enterocyte damage in septic shock patients: a prospective cohort study. Shock. (2018) 49:137–43. doi: 10.1097/shk.0000000000000955, 28786832

[6] Greis C Rasuly Z Janosi RA Kordelas L Beelen DW Liebregts T. Intestinal T lymphocyte homing is associated with gastric emptying and epithelial barrier function in critically ill: a prospective observational study. Crit Care. (2017) 21:70. doi: 10.1186/s13054-017-1654-9, 28327177 PMC5361812

[7] Bakshi J Mishra KP. Identification of biomarkers for gastrointestinal barrier injury and protective role of sodium butyrate in hypobaric hypoxia exposed rats. Int Immunopharmacol. (2025) 165:115424. doi: 10.1016/j.intimp.2025.115424, 40882548

[8] Bachmann KF Cotoia A Reintam BA. Gastrointestinal function and nutritional interventions in septic shock. Curr Opin Crit Care. (2025) 31:599–607. doi: 10.1097/mcc.0000000000001302, 40637059 PMC12419005

[9] Wang J Gao YL Yu WW Xia YH Sun YZ. Clinical significance of acute gastrointestinal injury grades in inflammatory response of critically ill patients. Zhonghua Yi Xue Za Zhi. (2017) 97:3312–5. doi: 10.3760/cma.j.issn.0376-2491.2017.42.008, 29141376

[10] Mao SH Feng DD Wang X Zhi YH Lei S Xing X . Magnolol protects against acute gastrointestinal injury in sepsis by down-regulating regulated on activation, normal T-cell expressed and secreted. World J Clin Cases. (2021) 9:10451–63. doi: 10.12998/wjcc.v9.i34.10451, 35004977 PMC8686136

[11] Lee SH. Intestinal permeability regulation by tight junction: implication on inflammatory bowel diseases. Intest Res. (2015) 13:11–8. doi: 10.5217/ir.2015.13.1.11, 25691839 PMC4316216

[12] Xiao YT Yan WH Cao Y Yan JK Cai W. Neutralization of Il-6 and Tnf-α ameliorates intestinal permeability in Dss-induced colitis. Cytokine. (2016) 83:189–92. doi: 10.1016/j.cyto.2016.04.012, 27155817

[13] Reintam Blaser A Malbrain ML Starkopf J Fruhwald S Jakob SM de Waele J . Gastrointestinal function in intensive care patients: terminology, definitions and management. Recommendations of the Esicm working group on abdominal problems. Intensive Care Med. (2012) 38:384–94. doi: 10.1007/s00134-011-2459-y, 22310869 PMC3286505

[14] Li H Zhang D Wang Y Zhao S. Association between acute gastrointestinal injury grading system and disease severity and prognosis in critically ill patients: a multicenter, prospective, observational study in China. J Crit Care. (2016) 36:24–8. doi: 10.1016/j.jcrc.2016.05.001, 27546743

[15] Zhonghua. Guidelines for medical nutritional therapy in adult sepsis patients (2025 edition). Zhonghua Yi Xue Za Zhi. (2025) 105:510–28. doi: 10.3760/cma.j.cn112137-20240813-01859 39956626

[16] Vittinghoff E Mcculloch CE. Relaxing the rule of ten events per variable in logistic and cox regression. Am J Epidemiol. (2007) 165:710–8. doi: 10.1093/aje/kwk052, 17182981

[17] Osterbur K Mann FA Kuroki K DeClue A. Multiple organ dysfunction syndrome in humans and animals. J Vet Intern Med. (2014) 28:1141–51. doi: 10.1111/jvim.12364, 24773159 PMC4857933

[18] Liang Y Ma C Ma T Lin F Guo T. The association between acute gastrointestinal injury and mortality in elderly patients with gram-positive bacterial bloodstream infection in the intensive care unit: a retrospective 7-year study from a research hospital in China. Front Med (Lausanne). (2025) 12:1634980. doi: 10.3389/fmed.2025.1634980, 41064509 PMC12500591

[19] Sun J Zhang J Wang X Ji F Ronco C Tian J . Gut-liver crosstalk in sepsis-induced liver injury. Crit Care. (2020) 24:614. doi: 10.1186/s13054-020-03327-1, 33076940 PMC7574296

[20] Zahs A Bird MD Ramirez L Turner JR Choudhry MA Kovacs EJ. Inhibition of long myosin light-chain kinase activation alleviates intestinal damage after binge ethanol exposure and burn injury. Am J Physiol Gastrointest Liver Physiol. (2012) 303:G705–12. doi: 10.1152/ajpgi.00157.2012, 22790598 PMC3468533

[21] Ji M Liang H Dong S Guo Y Lin Y Zhang H . The Swi/Snf chromatin-remodeling subunit Dpf2 regulates macrophage inflammation in intestinal injury via the Cacna1D-mediated Mapk pathway. Proc Natl Acad Sci U S A. (2025) 122:e2518762122. doi: 10.1073/pnas.2518762122, 41223220 PMC12646317

[22] Hu CH Chen Y Jin TY Wang Z Jin B Liao J . A derivative of tanshinone Iia and salviadione, 15a, inhibits inflammation and alleviates Dss-induced colitis in mice by direct binding and inhibition of Ripk2. Acta Pharmacol Sin. (2025) 46:672–86. doi: 10.1038/s41401-024-01399-1, 39443729 PMC11845706

[23] Bamias G Cominelli F. Cytokines and intestinal inflammation. Curr Opin Gastroenterol. (2016) 32:437–42. doi: 10.1097/mog.0000000000000315, 27673380

[24] Sun JK Huangfu XT Yin X Nie S Deng YH Ye ZY . Ferroptosis is involved in the Il-9-induced intestinal barrier injury in sepsis: an experimental animal and translational study. Int J Surg. (2025) 111:4314–24. doi: 10.1097/js9.0000000000002541, 40391960

[25] Guan X Chen D Xu Y. Clinical practice guidelines for nutritional assessment and monitoring of adult ICU patients in China. J Intensive Med. (2024) 4:137–59. doi: 10.1016/j.jointm.2023.12.002, 38681796 PMC11043647

[26] Bischoff SC Barbara G Buurman W Ockhuizen T Schulzke JD Serino M . Intestinal permeability--a new target for disease prevention and therapy. BMC Gastroenterol. (2014) 14:189. doi: 10.1186/s12876-014-0189-7, 25407511 PMC4253991

[27] Wang Y Li Y Wang H Li H Li Y Zhang L . Development and validation of a nomogram for predicting enteral feeding intolerance in critically ill patients (Nofi): mixed retrospective and prospective cohort study. Clin Nutr. (2023) 42:2293–301. doi: 10.1016/j.clnu.2023.10.003, 37852023

[28] Hu B Sun R Wu A Ni Y Liu J Guo F . Severity of acute gastrointestinal injury grade is a predictor of all-cause mortality in critically ill patients: a multicenter, prospective, observational study. Crit Care. (2017) 21:188. doi: 10.1186/s13054-017-1780-4, 28709443 PMC5513140

[29] Reintam Blaser A Padar M Mändul M Elke G Engel C Fischer K . Development of the gastrointestinal dysfunction score (Gids) for critically ill patients–a prospective multicenter observational study (isofa study). Clin Nutr. (2021) 40:4932–40. doi: 10.1016/j.clnu.2021.07.015, 34358839

[30] Soranno DE Coopersmith CM Brinkworth JF Factora FNF Muntean JH Mythen MG . A review of gut failure as a cause and consequence of critical illness. Crit Care. (2025) 29:91. doi: 10.1186/s13054-025-05309-7, 40011975 PMC11866815

