# Supplementary material for: The role of cytokines in acute gastrointestinal injury: a prospective pilot study
Source: Front Med (Lausanne). 2026 Mar 4;13:1701855. doi: 10.3389/fmed.2026.1701855 (PMC12996046; doi:10.3389/fmed.2026.1701855)
Supplement: Supplementary file 1 [file Data_Sheet_1.pdf]

The Role of Cytokines in Acute Gastrointestinal Injury: A Prospective Pilot Study

Yanhua Li [a], Youquan Wang [a], Lu Ke[b], Xinyu Li [a], Lingling Bao [a], Feng Zhang [a], Hongxiang Li[a*], Dong Zhang [a*]

[a] *Department of Critical Care Medicine, The First Hospital of Jilin University, Changchun, 130021, China*

[b] *Department of Critical Care Medicine, Jinling Hospital, Medical School of Nanjing University, Nanjing 210002, Jiangsu, China; National Institute of Healthcare Data Science, Nanjing University, Nanjing 210010, Jiangsu, China*

Corresponding Author:

Hongxiang Li, Email: li_hx@jlu.edu.cn

Dong Zhang, Email: zhangdong@jlu.edu.cn

Additional emails:

Yanhua Li email: liyanhua@jlu.edu.cn

Youquan Wang email: wangyq20@mails.jlu.edu.cn

Lu Ke email: kelu@nju.edu.cn

Xinyu Li email: xinyuli@jlu.edu.cn

Lingling Bao: baoll24@mails.jlu.edu.cn

Feng Zhang email: zhangfengmodest@163.com

Supplementary Information

Supplementary Table S1 Classification of AGI

Grade	Definition
I (risk of GI dysfunction or failure)	Partial impairment of GI function, manifested as gastrointestinal symptoms related to a known cause and perceived to be transient. Examples: postoperative nausea and/or vomiting during the first few days after abdominal surgery, postoperative absence of bowel sounds, diminished bowel motility in the early phase of shock.
II (GI dysfunction)	The GI tract is unable to perform digestion and absorption adequately to satisfy the nutrient and fluid requirements of the body. There are no changes in the general condition of the patient due to GI problems. Examples: gastroparesis with high gastric residuals or reflux, paralysis of the lower GI tract, diarrhea, intra-abdominal pressure (IAP) 12–15 mmHg, visible blood in gastric content or stool. Feeding intolerance is present if at least 20 kcal/kg BW/day via the enteral route cannot be achieved within 72 h of a feeding attempt.
III (GI failure)	Loss of GI function. Restoration of GI function is not achieved despite interventions, and the general condition is not improving. Examples: persistent feeding intolerance despite treatment manifested as high gastric residuals, persistent GI paralysis, occurrence or worsening of bowel dilatation, IAP, 15–20 mmHg, low abdominal perfusion pressure (below 60 mmHg). Feeding intolerance is present and possibly associated with persistence or worsening of multiple organ dysfunction syndrome.
IV (GI failure with severe impact on distant organ function)	AGI has progressed to become directly and immediately life-threatening, with worsening of multiple organ dysfunction syndrome and shock. Examples: bowel ischemia with necrosis, GI bleeding leading to hemorrhagic shock, Ogilvie syndrome, abdominal compartment syndrome requiring decompression.

Primary AGI is associated with primary disease or direct injury to organs of the GI system, such as peritonitis, pancreatitis, abdominal surgery. Secondary AGI develops as a consequence of the host response to critical illness without a primary pathology in the GI system, such as GI malfunction in a patient with pneumonia or non-abdominal surgery

AGI acute gastrointestinal injury, BW body weight, GI gastrointestinal, IAP intra-abdominal pressure

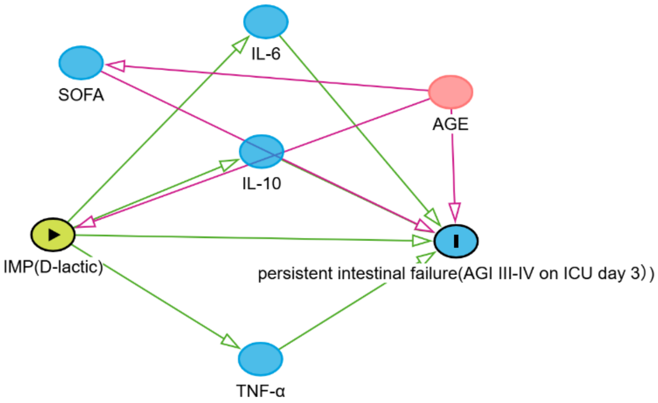

Supplementary Figure S1 Directed acyclic graph for the causal mediation framework linking intestinal permeability to persistent intestinal failure via inflammatory cytokines.

SOFA, Sequential Organ Failure Assessmen; Interleukin; TNF, tumor necrosis factor; IMP, Intestinal mucosal permeability; AGI acute gastrointestinal injury

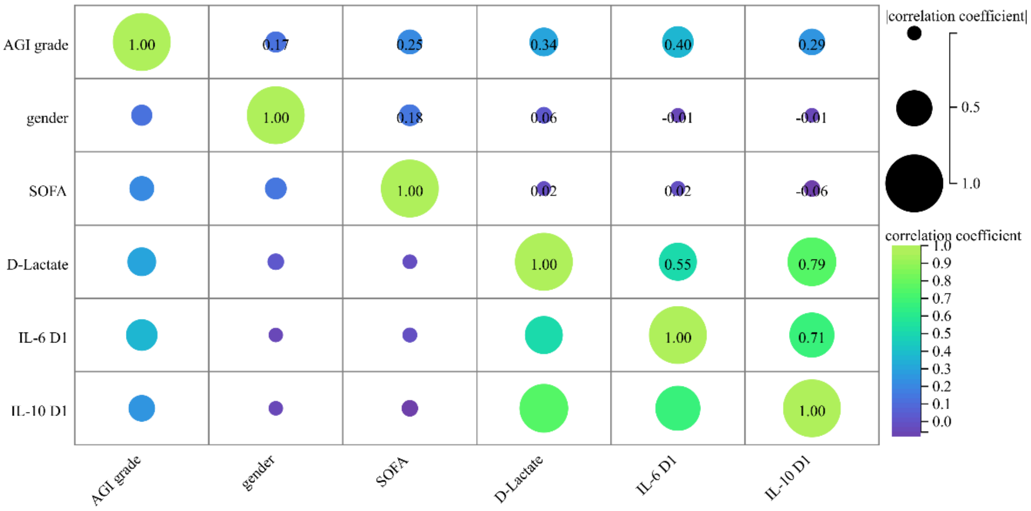

Supplementary Figure S2. Correlation analysis between variables in the Logistic Regression Analysis

Supplementary Table S2 Reference values of plasma IL-6, IL-10, and TNF-α in healthy individuals

Biomarker	Median (IQR, 25%–75%)
IL-6(pg/ml)	2.64(1.23-4.01)
IL-10 (pg/ml)	5.68(3.23-7.04)
TNF-α(pg/ml)	6.45(2.36-9.34)

IL, Interleukin; TNF, tumor necrosis factor;

Values were obtained from 50 healthy volunteers recruited from the Health Checkup Center of the First Hospital of Jilin University (men, 56%), with a mean age of 47.9 ± 15.1 years.

Supplementary Table S3: Results of mediation analysis

Model	Mediator1 IL-6				Mediator 2 IL-10				Mediator 3 TNF-α			
D Lactic acid→AGI grade	Effect Estimate	SE	95% CI	p-value	Effect Estimate	SE	95% CI	p-value	Effect Estimate	SE	95% CI	p-value
a (exposure mediator)	0.620	0.122	0.392-0.855	< 0.001	0.441	0.032	0.378-0.503	< 0.001	0.828	0.066	0.726-1.095	< 0.001
b (mediator outcome)	0.013	0.051	0.003-0.035	0.010	0.013	0.003	0.008-0.018	< 0.001	0.004	0.001	0.002-0.007	0.001
c (total effect)	0.011	0.001	0.001-0.023	0.007	0.011	0.001	0.001-0.023	0.007	0.011	0.001	0.001-0.023	0.007
c' (direct effect)	0.003	0.010	0.001-0.032	0.026	0.006	0.002	0.002-0.035	0.026	0.010	0.001	0.001-0.031	0.026
ab (mediated effect)	0.008	0.134	0.001-0.126	0.044	0.006	0.006	0.002-0.125	0.041	0.003	0.418	0.002-0.096	0.035
percentage mediated %	73.3%				52.1%				30.1%			
